# OvAge: a new methodology to quantify ovarian reserve combining clinical, biochemical and 3D-ultrasonographic parameters

**DOI:** 10.1186/s13048-015-0149-z

**Published:** 2015-04-08

**Authors:** Roberta Venturella, Daniela Lico, Alessia Sarica, Maria Pia Falbo, Elio Gulletta, Michele Morelli, Errico Zupi, Gabriele Cevenini, Mario Cannataro, Fulvio Zullo

**Affiliations:** Unit of Obstetrics and Gynecology, Magna Graecia University of Catanzaro, Viale Europa - Localitá Germaneto, 88100 Catanzaro, Italy; School of Informatics and Biomedical Engineering-Bioinformatics Laboratory, Magna Graecia University of Catanzaro, Viale Europa - Localitá Germaneto, 88100 Catanzaro, Italy; Chair of Clinical Pathology, Magna Graecia University of Catanzaro, Viale Europa - Localitá Germaneto, 88100 Catanzaro, Italy; Department of Molecular and Developmental Medicine, University of Siena, Siena, Italy; Department of Medical Biotechnologies, University of Siena, Siena, Italy

**Keywords:** Ovarian reserve, Follicle Stimulating Hormone, Anti Müllerian Hormone, 3D Antral Follicle Count, Distance to menopause, Reproduction, Family planning, Generalized linear model

## Abstract

**Background:**

In the last decade, both endocrine and ultrasound data have been tested to verify their usefulness for assessing ovarian reserve, but the ideal marker does not yet exist. The purpose of this study was to find, if any, a statistical advanced model able to identify a simple, easy to understand and intuitive modality for defining ovarian age by combining clinical, biochemical and 3D-ultrasonographic data.

**Methods:**

This is a population-based observational study. From January 2012 to March 2014, we enrolled 652 healthy fertile women, 29 patients with clinical suspect of premature ovarian insufficiency (POI) and 29 patients with Polycystic Ovary syndrome (PCOS) at the Unit of Obstetrics & Gynecology of Magna Graecia University of Catanzaro (Italy). In all women we measured Anti Müllerian Hormone (AMH), Follicle Stimulating Hormone (FSH), Estradiol (E2), 3D Antral Follicle Count (AFC), ovarian volume, Vascular Index (VI) and Flow Index (FI) between days 1 and 4 of menstrual cycle. We applied the Generalized Linear Models (GzLM) for producing an equation combining these data to provide a ready to use information about women ovarian reserve, here called OvAge. To introduce this new variable, expression of ovarian reserve, we assumed that in healthy fertile women ovarian age is identical to chronological age.

**Results:**

GzLM applied on the healthy fertile controls dataset produced the following equation OvAge = 48.05 - 3.14*AHM + 0.07*FSH - 0.77*AFC - 0.11*FI + 0.25*VI + 0.1*AMH*AFC + 0.02*FSH*AFC. This model showed a high statistical significance for each marker included in the equation. We applied the final equation on POI and PCOS datasets to test its ability of discovering significant deviation from normality and we obtained a mean of predicted ovarian age significantly different from the mean of chronological age in both groups.

**Conclusions:**

OvAge is one of the first reliable attempt to create a new method able to identify a simple, easy to understand and intuitive modality for defining ovarian reserve by combining clinical, biochemical and 3D-ultrasonographic data. Although design data prove a statistical high accuracy of the model, we are going to plan a clinical validation of model reliability in predicting reproductive prognosis and distance to menopause.

**Electronic supplementary material:**

The online version of this article (doi:10.1186/s13048-015-0149-z) contains supplementary material, which is available to authorized users.

## Background

The normal process of reproductive ageing varies considerably among women. While some women undergo premature menopause, others have spontaneous pregnancy in their fourth decade of life [1]. This variability, currently not predictable, is summarized in the concept of ovarian reserve, a term coined more than 25 years ago by Daniel Navot [2] and present in titles of at least 1300 papers indexed on PubMed.

Given this variability, the ability to predict the ovarian reserve both in terms of reproductive prognosis and distance to menopause would be extremely important for any modern physician and gynecologist, in guiding patients’ reproductive attempts and in reducing the rates of unnecessary surgeries for benign pathologies, for which menopause may represent the best therapy.

Up to now, no test, neither single nor combined, possesses a satisfactory sensitivity and specificity for clinical application [3]. The explanation to this incompetence probably lies in the conundrum of any screening test for ovarian reserve insufficiency: its prevalence changes with age and the performance of any test changes with it [4]. This concept explains why no single threshold value is usable to define an impairment of ovarian reserve, and why every test should always be interpreted based on the chronological patient’s age.

The ideal marker should be able to evaluate simultaneously the qualitative and quantitative aspects of ovarian reserve, to integrate all the relevant information and respond with an output of clear understanding, that we could name “ovarian age”. Every woman could easily compare this number with her chronological age and the general practitioner would understand its meaning without being an expert in reproductive medicine.

With this purpose, we introduce the new concept of OvAge, a numeric variable, which accurately reflects the ovarian reserve of women. Since the ovarian age is inherently unknown, we assumed that OvAge coincides with the chronological age, in healthy fertile women. A large representative sample of the healthy fertile women population has been used to design a statistical model able to estimate OvAge by a mathematical formula which accounts for patient’s biochemical and ultrasonographic as predictor variables. Biochemical variables include Follicle Stimulating Hormone (FSH), Estradiol (E_2_), Anti Müllerian Hormone (AMH), 3D-ultrasonographic variables are Antral Follicle Count (AFC), ovarian volume, Flow index (FI), Vascularization Index (VI) and Vascularization Flow Index (VFI). We designed a Generalized Linear Model (GzLM) and we finally used the model to evaluate the clinical plausibility of OvAge estimations in women with pathologic derangements of ovarian age, like patients with premature ovarian insufficiency (POI) and polycystic ovary syndrome (PCOS). In particular, the increase or decrease of OvAge from chronological age were interpreted as a useful clinical index of physiopathological ovarian reserve.

## Materials and methods

This is a population-based study including the data of South Italy women enrolled after a delivery following a spontaneous pregnancy, or during a routine visit for cervical screening or contraception counselling at the Unit of Obstetrics & Gynecology and analyzed in collaboration with the Unit of Clinical Pathology and the School of Informatics and Biomedical Engineering, at Magna Graecia University of Catanzaro, Italy.

The study was approved by the local Ethics Committee and written, informed consensus was obtained from each woman to the anonymous use of clinical data for statistical evaluation and research purposes.

From January 2012 to March 2014 we consecutively recruited women, aged 18 to 55, who consent to participate in this prospective study, with history of spontaneous conception(s), intact ovaries and regular menses with a mean interval of 21 to 35 days.

Exclusion criteria for the selection of the training subjects for this model were: estrogen or progestin use or in breastfeeding the two months before enrollment, pregnancy, history of female infertility, endometriosis, presence of ovarian follicles measuring more than 10 mm at study entry ultrasonography and other cystic masses of the ovary, history of ovarian surgery, PCOS, gynecological malignancy, previous radiation or chemotherapy, autoimmune disease, known chronic, systemic, metabolic and endocrine disease including hyperandrogenism, hyperprolactinemia, diabetes mellitus and thyroid diseases, hypogonadotropic hypogonadism or with history of use of drugs that can cause menstrual irregularity.

During the same enrollment period, women with clinical suspect of POI and women with PCOS were also enrolled, to test the ability of OvAge to detect abnormal deviation from chronological age.

POI was defined as the presence of secondary amenorrhea before the age of 40 years associated to FSH value >30 U/L and clinical manifestations such as palpitations, flushes, fatigue, anxiety, depression [5]. PCOS was diagnosed according to the Rotterdam criteria [6], when at least 2 of the following 3 features existed: oligo/amenorrhea, clinical and/or biochemical hyperandrogenism, and polycystic ovaries morphology (PCOM). We chose to include only PCOS women with PCOM to test our algorithm in this particular population of patients because, notwithstanding the lack of absolute evidence regarding dissimilarities involved in regulation of ovarian ageing in these women, data currently available in literature suggest that the intrinsic ovarian abnormality associated with altered follicular dynamics in the PCOS might cause a reduced rate of atresia, a delayed age of menopause and end of fertility [7].

At screening visit, all women were checked for inclusion and exclusion criteria by anamnesis and clinical examination. Anamnesis was collected by a standardized questionnaire aimed to investigate also past problems in conceiving and menstrual disorders.

Eligible women were asked to contact one of the investigators at the first day of menstrual cycle, at least three to four months after delivery.

At study entry, basal AMH, FSH and E_2_, AFC, ovarian volume, VI, FI and VFI were measured in all women between day 1 and day 4 of menstrual cycle.

### Hormonal assays

Blood samples obtained by venipuncture were centrifuged, within 30 minutes from the collection, for 10 min at 3500 rpm, 4°C. Aliquots of each serum were frozen at −80°C and stored for subsequent assays of AMH, FSH and E2. To measure serum AMH levels, AMH-Gen II ELISA assay kit (Beckman Coulter) was used. The lowest detection limit of AMH is 0.08 ng/mL and the intra and interassay coefficients of variation below 3.4% and 4.0% respectively. The ECLIA method used to measure the levels of serum FSH and E2 by COBAS e411 auto-analyser (Roche Diagnostics). The lowest detection limit for FSH is 0.100 IU/L, with the intra- and interassay coefficients of variation below 2.6% and 3.5% respectively. The lowest detection limit for E2 is 18.4 pmol/L, with the intra- and interassay coefficients of variation of 2% and 3%, respectively.

Giving the poor reliability of the FSH value in presence of Estradiol levels greater than 70 pg/mL, women with basal E2 levels greater than this cut off were asked to come back at the beginning of the subsequent menstrual cycle, and their data were excluded from the analysis.

### Ultrasonographic data collection and post-processing

The same day of hormonal assay, a single experienced investigator (DL) performed all the ultrasound scans using a Voluson-i (GE Healthcare Ultrasound) and a 5–9-MHz transvaginal volume transducer, which has 3D ultrasound scanning modes. Antral follicle number and vascularization indices were measured using a 3D ultrasound dataset, with Sonography-based Automated Volume Count and Virtual Organ Computer Aided Analysis Imaging Program (SonoAVC™ and VOCA™, GE Healthcare Ultrasound, Zipf, Austria), as previously described by other authors [8].

The acquired 3D ultrasound datasets were displayed in the multiplanar view. The image were optimized to generate a three-dimensional volume of interest (VOI) and to ensure that the whole ovary was included without extra-ovarian information.

SonoAVC was applied for automatically identifying and quantifying hypoechoic areas within a 3D ultrasound dataset. Post-processing, involving the manual identification of follicles not included in the previous automated analysis, was then used to ensure that all antral follicles were counted. The total antral follicle count for each subject was recorded to the nearest millimeter, starting from 2.0 mm up to a maximum of 10.0 mm. Patients with ovarian follicles greater than 10 mm were asked to come back at the beginning of the subsequent menstrual cycle.

The ultrasound machine was switched to the 3D mode with power Doppler. The setting condition for this study was as follows: frequency mid; dynamic set 2; balance G >140; smooth 5/5; ensemble 12; line density 7; power Doppler map 5, sweep angle 30°. The setting condition for the sub-power Doppler mode was as follows: gain 6.0; balance 140; quality normal; wall motion filter low 1; velocity range 0.9 kHz. A 3D dataset was then acquired using the medium speed sweep mode. The built-in VOCAL (virtual organ computer aided analysis) Imaging Program for the 3D power Doppler histogram analysis was used to determine the ovarian volume and indices of vascularization and blood flow. Vascularization index (VI) measured the number of blood vessels in the ovary (colour voxels) and was expressed as a percentage (%) of the ovarian volume. Flow index (FI) represented the average intensity of flow inside the ovary and Vascularization flow index (VFI), made by multiplying VI and FI, was a combination of vascularization and flow. During the analysis and calculation, the manual mode of the VOCAL Contour Editor was used to cover the whole 3D volume of the ovary with a 15° rotation step. Hence, 12 contour planes were analyzed for each ovary to cover 180°.

The intra-observer reliability was expressed as the mean intra-class correlation coefficient (ICC) with 95% confidence interval (CI). The mean ICC (95% CI) for 3D scanning of ovarian volume, VI, FI and VFI were 0.9709 (0.8692, 0.9837), 0.9796 (0.9659, 0.9876), 0.8876 (0.7143, 0.9723) and 0.9934 (0.9761, 0.9987) respectively. The mean ICC for data acquisition of ovarian volume, VI, FI and VFI were 0.9945 (0.9745, 0.9923), 0.9845 (0.9432, 0.9954), 0.9878 (0.9621, 0.9954) and 0.9865 (0.9523, 0.9967) respectively.

### Statistical analysis

A dataset of 710 records of subjects has been analyzed using Konstanz Information Miner (KNIME) v.2.9.2 and KNIME R Statistics Integration v.2.9.1 for integrating R language v.3.0.3.

The whole analysis consists in six main steps: (i) definition and creation of three datasets, Healthy Controls, POIs and PCOS; (ii) descriptive statistics and statistical analysis of datasets and construction of growth curves centile by the LMS method [9]; (iii) generation of Generalized Linear Models (GzLMs); (iv) choice of best model by stepwise selection, according to the Aikake Information Criterion (AIC) [10]; (v) reconstruction and refinement of the best GzLM model; (vi) evaluation and testing of the final model; (vii) application of the model on POI and PCOS datasets.

For details of statistical methods, see the Additional file 1.

## Results

From January 2012 to March 2014, 840 women were screened for the enrollment. According to exclusion and inclusion criteria, 96 patients were not considered eligible and other 34 refused to participate, hence a total of 710 women were enrolled and analyzed.

**Figure 1:**
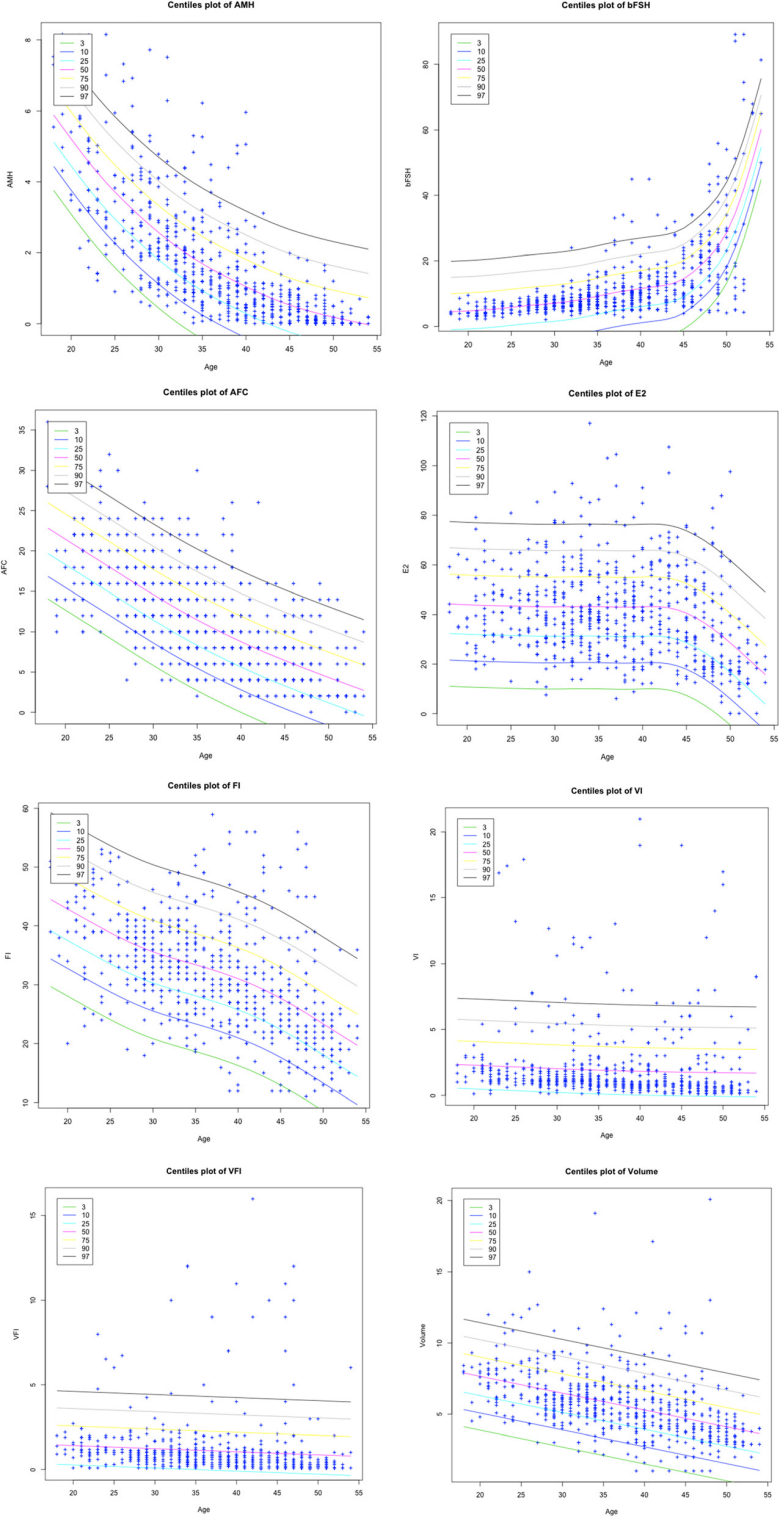
**Plots of growth curve centile (3th, 10th, 25th, 50th, 75th, 90th, 97th) for each feature where Age is the covariate.** The LMS method and penalized likelihood is used for fitting distributions.

Table 1 summarizes the statistics of healthy controls (HC, 652 subjects), POIs (29 subjects) and PCOS (29 subjects) respectively, for age, parity, AMH, FSH, E_2_, AFC, FI, VI, VFI and Volume. Spearman’s test coefficients showed statistical dependences between Age and AMH (−0.8090), FSH (0.6742), E_2_ (−0.2289), AFC (−0.7304), FI (−0.5649), VI (−0.4066), VFI (−0.3428) and Volume (−0.5519). Holm’s method corroborated this correlation, providing significant adjusted p-values (p < 0.0001) for each dependent variable. In Figure 1 the centile curves are depicted for each feature.

**Table 1:** **Descriptive statistics for healthy control subjects (652 subjects), POI affected subjects (29 subjects) and PCOS affected subjects (29 subjects)**

	**Healthy controls (652 subjects)**	**POI subjects (29 subjects)**	**PCOS subjects (29 subjects)**
Age *[years]*	36.80 ± 8.62	37.90 ± 3.31	27.45 ± 4.30
Parity *[number]*	1.37 ± 0.97	0.44 ± 0.57	1.21 ± 0.67
AMH *[ng/ml]*	1.74 ± 1.75	0.01 ± 0.04	7.19 ± 2.13
FSH *[mlU/ml]*	12.47 ± 11.94	59.37 ± 23.52	6.45 ± 1.44
E_2_ *[pg/ml]*	40.76 ± 18.56	26.24 ± 17.02	49.90 ± 15.53
AFC	10.97 ± 6.71	3.24 ± 2.23	28.90 ± 6.51
FI	32.16 ± 9.46	22.38 ± 6.94	38.14 ± 6.35
VI	1.91 ± 2.68	1.46 ± 2.97	1.81 ± 0.98
VFI	1.11 ± 1.70	0.63 ± 0.85	1.26 ± 0.70
Volume	5.66 ± 2.25	3.72 ± 1.76	7.72 ± 1.98

Data are expressed as mean ± SD.

Table 2 presents a summary of the ten models according to the predictors chosen for the equation and reports AIC, the testing errors evaluated through the leave-one-out (LOO) and cross-validation technique.

**Table 2:** **Comparison of Generalized Linear Models in term of AIC as the predictors change**

**Model**	**Predictor**	**AIC**
GzLM#1	AMH + FSH + E_2_ + AFC + FI + VI + VFI + Volume	3987.3
GzLM#2	AMH + FSH + AFC + FI + VI	3981.9
GzLM#2*	AMH + FSH + AFC + FI + VI + AMH*AFC + FSH*AFC	3955.2
GzLM#3	AMH	4175.5
GzLM#4	AMH + FSH	4052.1
GzLM#5	AMH + FSH + E_2_	4053.8
GzLM#6	AMH + FSH + AFC	4003.4
GzLM#7	FI + VI + VFI + Volume	4449.9
GzLM#8	AFC	4215.6
GzLM#9	AFC + Volume	4190
GzLM#10	Volume	4651.7

In the first model, GzLM#1, only E_2_, VFI and Volume had shown p-values greater than 0.01. These three variables were then removed and a second model, GzLM#2, was created. In GzLM#2, all the five variables showed a *p-*value < 0.001, that together with the lowest AIC (and the highest leave-one-out and 10-fold cross-validation accuracy) leaded to accept this set of attributes as the best one.

The model GzLM#2* contains two more terms, AMH:AFC (*p*-value < 0.001) and FSH:AFC (*p-*value < 0.001). GzLM#2* showed a lower AIC value (3955.2) compared to the previous models. ANOVA test was performed for comparing model GzLM#2* to the null model: a p-value < 0.001 for AMH, FSH, AFC, FI, AHM:AFC, FSH:AFC and a *p*-value < 0.01 for VI, successfully confirmed the prediction power of the variables.

The goodness of fitness of the model GzLM#2* was first evaluated by a Chi-squared test obtaining a p-value of 1, which was large enough to indicate no evidence of a lack of fit.

**Figure 2:**
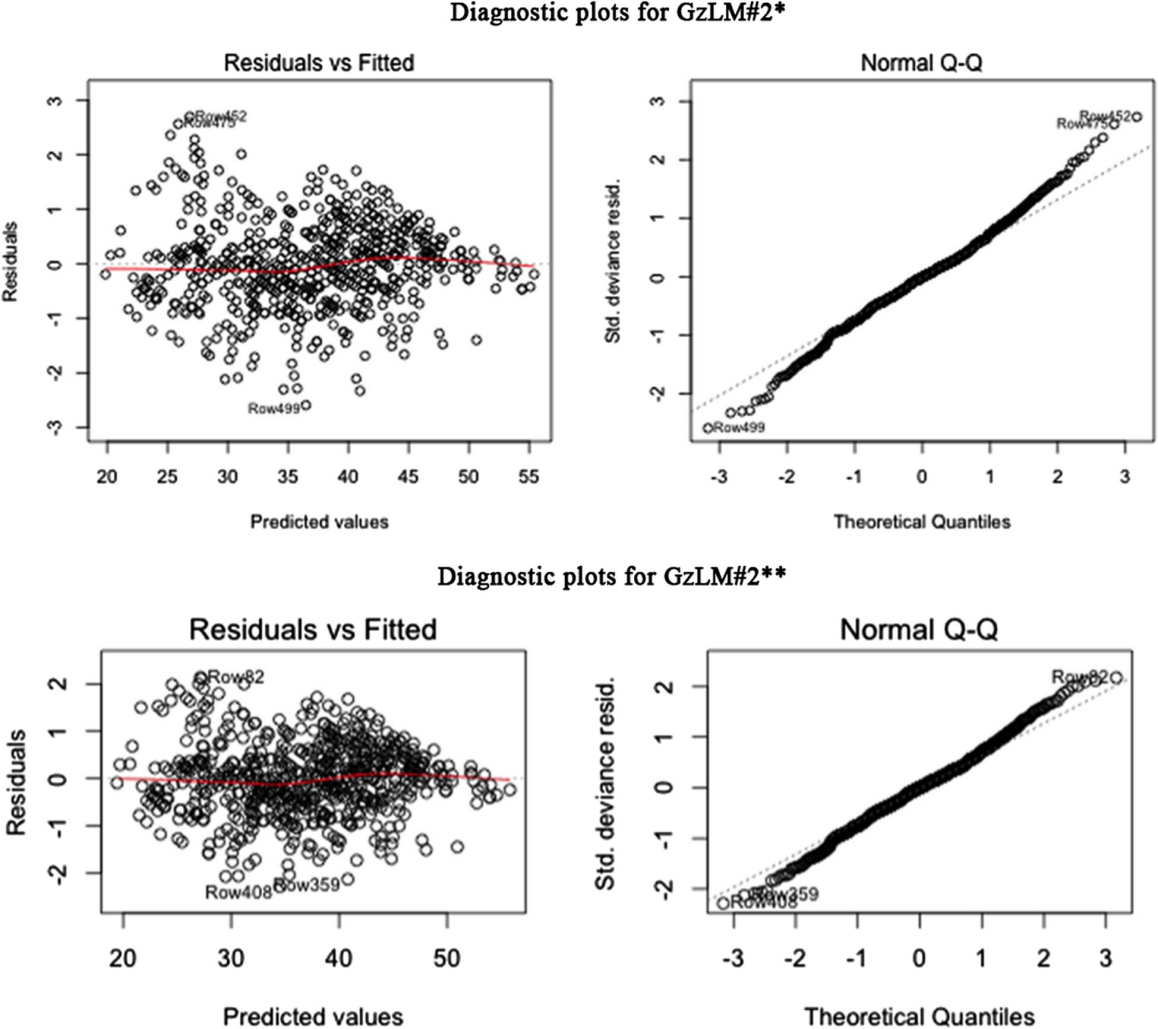
Diagnostic plots for (a) model GzLM#2* and (b) model GzLM#2**. On the left Pearson residuals plotted against predicted values and on the right the Normal Q-Q plot. The red line in the plot of residuals of GzLM#2** presents a slight improvement in the linear trend compared to the plot of model GzLM#2*.

In the upper part of the Figure 2a, diagnostic plots for model GzLM#2* are depicted. In the plot on the left, Pearson residuals were plotted against predicted values. The red line almost coincides with the horizontal one proving that the constant variance assumption on the errors is verified and thus that the model shows a high level of fit.

The Normal Q-Q plot on the right of the upper part of Figure 2a shows that dots follow a linear trend even if several cases at the ends influence the normality. For this reason, the model, now called GzLM#2**, has been updated without influence cases. Seven cases in total have been removed from the final dataset and then analyzed by specialists in order to search for possible abnormalities. Such further investigation showed that those patients were not affected by any pathology and the only reason for excluding them is related to their high Cook’s distance, that is the measure of influence on the regression coefficients.

The bottom part of the Figure 2b depicts diagnostic plots for the new GzLM#2** model. The Normal Q-Q plot still shows several cases diverge from linearity but their distances are acceptable, compared to the Normal Q-Q plot of GzLM#2*. The red line in the plot of residuals against the fitted values of GzLM#2** (on the left), presents a slight improvement in the linear trend compared to the plot of model GzLM#2*.

The LOO and ten-fold cross-validation accuracy for GzLM#2** are respectively 79.865% and 79.832%, with an improvement of about 3% of accuracy compared to the previous models.

For these reasons, we can certainly assess that in our dataset of 645 healthy controls, the best equation for predicting the ovarian age is: OvAge = 48.05-3.14*AHM + 0.07*FSH-0.77*AFC-0.11*FI + 0.25*VI + 0.1*AMH*AFC + 0.02*FSH*AFC.

GzLM#2** has been applied on POI and PCOS datasets to test its power of discovering significant deviation from normality. The ovarian age of each POI subject resulted to be, as expected, significantly higher than the chronological age. The mean of their OvAge is indeed 50.63 ± 3.80 years, against a mean of chronological age of 37.90 ± 3.31 years.

Regarding PCOS subjects, the mean OvAge (24.98 ± 0.91 years) of 24 PCOS subjects resulted to be significantly lower than the chronological age (29 ± 2.75 years).

## Discussion

In this prospective study, we collected data of clinical, biochemical and 3D-ultrasonographic parameters in a population of healthy fertile women with the aim of developing a new methodology to quantify ovarian age (OvAge). To introduce this new variable, expression of ovarian reserve, we assumed that in healthy women with proven fertility ovarian age corresponds to chronological age.

In women enrolled as healthy fertile controls, our new model showed a high level of fit between chronological age and predicted OvAge. In POIs and PCOS patients, conversely, a significant difference between these two parameters was shown, indicating that the formula produced is able to recognize pathological deviation from normal ovarian function.

Moreover, in all 18 women with a predicted ovarian age between 49 and 51 years at enrollment, regardless of their chronological age, menopause occurred within 1–2 years during the study period (data not shown).

Although preliminary, these results are very promising and drive us to further investigations and to a follow-up clinical validation of our model. The prospective evaluation of distance to menopause in our population, for example, is ongoing, and our statisticians are working to reach quickly the highest possible predictive ability, by means of accelerated failure time modeling. This approach was recently used by Tehrani et al. [11], but their analysis was conducted by measuring AMH, collected on any days of the menstrual cycles, and they did not use other markers of ovarian ageing [11]. The evaluation of our 18 cases in which OvAge was more reliable than chronological age in determining the time to menopause, however, is already a first clinical confirmation of its reliability.

With OvAge, we aim to answer the need of any modern gynecologist to find the ideal marker of ovarian function. It should be universally accepted, reproducible, easy to interpret and applicable to the general population, and has to be able to give reliable information on number and quality of follicles, residual fertility, time to menopause and reproductive prognosis, both natural and post-Assisted Reproductive Techniques (ART).

Basal serum FSH, extensively studied over the past few decades, only provides a crude surrogate marker of biological ovarian age when paired with the chronological age but it is probably the most indirect marker of ovarian reserve to date [12]. Moreover, FSH variability between follicular phases has been reported to be above 50% of the measured value, thus inevitably reducing the global predictive performance of this marker [13].

Recent studies have suggested that serum AMH levels represent ovarian quantitative reserve in IVF patients and may provide an index of age at menopause [11,14,15]. Thanks to its ability of providing valuable information regardless of what day in the cycle the sample is drawn and in women who do not menstruate, AMH has gained popularity among infertility specialists. In a recent retrospective study on women undergoing their first IVF/ICSI cycle, however, concordance between AMH and FSH was noted in 57% of women while 43% of women had discordant values. According to these results, authors concluded that both AMH and FSH tests are probably useful to predict ovarian response but since many women have discordant values, it is difficult to counsel patients regarding their true ovarian reserve using one single marker [16].

In a recent meta-analysis, authors showed that even if AMH, independently of age, has some association with predicting live birth after assisted conception, its predictive accuracy is poor, concluding that no patient should be precluded from attempting ART solely on the basis of an AMH value [17]. Moreover, only very limited data on AMH and natural fertility at different stages of reproductive life are available today, since AMH levels have been extensively studied in infertile women but less in fertile ones.

With the recent acquisition of one of the two commercially available assays in commerce by Beckman-Coulter, the two existing kit were replaced by a new enzyme-linked immunosorbent assay. Recent safety notices, indicating the risk of falsely low and falsely elevated values due to technical mistakes, have fueled concerns about the robustness of the new assay [18]. This issue, once again, limits the actual possibility of considering AMH as the best single marker, sufficient by itself, for ovarian reserve assessment.

Studies on ultrasonographic parameters and ovarian reserve prediction reported similar conclusions. While AFC provides a direct estimation of recruitable follicles and gradually declines with chronological age, less data are today available on ovarian flow indexes [19].

According to the results of his meta-analysis, Gibreel in 2009 concluded that although Doppler studies of ovarian stromal blood flow are promising, more studies are warranted, also to determine the optimum parameters and the best cut-off values for these indices [20]. It is known, however, that three-dimensional Doppler technique yields better axial resolution, fewer blooming artifacts, and improved sensitivity to slow flows and small vessels compared with standard bi-dimensional power Doppler [21]. This advantage makes 3D angiography attractive since one speculative cause of age-related increase in oocyte chromosomal meiotic errors is a reduced oxygen levels in the follicular fluid caused by a compromised microcirculation around the leading follicle [22].

All these observations confirm the need of assessing ovarian age through dynamic multimodal tools able to improve the limited accuracy of the single parameter evaluated individually.

To the best of our knowledge, this is the first attempt to create a multimodal methodology able to answer with an easy-to-interpret response, the exact ovarian age or OvAge.

The developed equation combines complementary evaluations and gives as a result explicitly the ovarian age, a ready-to-use information, which will be useful for both women and physicians. In future, if it will be validated by our ongoing prospective evaluation, OvAge could provide reliable information to gynecologists for counseling patients in their reproductive attempts and for reducing the rates of unnecessary surgery in the management of benign pathologies, such as dysfunctional abnormal uterine bleeding, adenomyosis or fibromatous uterus, which may best be treated with medical therapy, if natural menopause will be predicted to occur within few years.

OvAge could help researchers in quantifying the effect of surgeries, medications and ovarian pathologies themselves on women’s ovarian in term of years of ageing, with the advantage of an intuitive, easily communicable and universal information available to both physicians and patients.

## Conclusions

In conclusion, to the best of our knowledge, this is the first attempt to build a mathematical formula in which, once single patient’s biochemical and ultrasonographic values are introduced as input, the generated output is an easy-to-interpret number, which reliably expresses the ovarian reserve of that woman. This formula and its output, both called OvAge, put together different markers with different meanings to increase the reliability of the new ovarian reserve parameter.

## Additional file

Additional file 1:
**Detailed statistical analysis.**

## Abbreviations

POI: Premature ovarian insufficiency
PCOS: Polycystic ovary syndrome
AMH: Anti Müllerian hormone
FSH: Follicle stimulating hormone
E2: Estradiol
AFC: 3D antral follicle count
VI: Vascular index
FI: Flow index
VFI: Vascular flow index
GzLM: Generalized Linear Models
