# Supplementary material for: OvAge: a new methodology to quantify ovarian reserve combining clinical, biochemical and 3D-ultrasonographic parameters
Source: J Ovarian Res. 2015 Apr 8;8:21. doi: 10.1186/s13048-015-0149-z (PMC4392473; doi:10.1186/s13048-015-0149-z)
Supplement: Additional file 1: — Detailed statistical analysis. [file 13048_2015_149_MOESM1_ESM.docx]

**Additional material**

**Detailed statistical analysis**

(i) The main dataset has been divided into three sub-datasets, according to the preliminary clinical analysis HCs (Healthy Controls), POI and PCOS. Each subset has been checked for assuring the data quality, in terms of the presence of missing values and consistency in decimal separator.

(ii) For each dataset, descriptive statistics have been calculated for each feature. A qualitative and quantitative evaluation of the correlation between the chronological age and the eight variables have been performed by applying Spearman’s test and Holm’s test for adjusted p-values.

(iii) For describing the relationship between the new response variable here introduced and called ovage, and the set of inputs, AMH, FSH, E2, AFC, FI, VI, VFI and Volume that are the independent variables, we applied the Generalized linear model (GzLM) [1] theory, which provides a unified methodology for modeling all types of response variables, such as continuous, binary, ordinal response or variables in the form of proportions. Since it has been hypothesized that, in healthy population, ovage is equal to chronological age, which is not continuous and non-normal, a Poisson distribution has been chosen as the *random component* of the GzLM for modeling the expected value of ovage. Identity has been chosen as a *link function*, and it has been preferred to the *log link* for taking into account the interactions between covariates [2] as described in step v.

(iv) For avoiding collinearity problems due to the possible non independence of predictor variables [3], a hybrid approach, using backward and forward selection together, has been applied. The overall best model was chosen using the Akaike information criterion (AIC) [4], which is considered a good estimator of predictive accuracy than the Bayesian Information Criterion (BIC). BIC tends indeed to underfit the data and to introduce bias of overestimating precision [5]. AIC is asymptotically equivalent to *K-fold cross-validation* in model selection and this is true for any model [6], and asymptotically, minimizing BIC is equivalent to leave–*v*-out cross-validation [7]. For the sake of completeness, for each generated model, 10-fold cross-validated accuracy and leave-one-out accuracy have been calculated too. In particular, in the 10-fold cross-validation, the dataset is divided into 10 groups of approximately equal size and for each group the generalized linear model is fit to nine of the groups (training set) omitting one group that is then used as test set. In the leave-one-out only one observation is left out as test set.

As further evidence of the significance of the linear terms, ANOVA test (Chi-squared) had been performed for comparing each model to the null model in terms of deviance.

(v) The best model had been re-built taking into account all possible interactions among covariates to assess any improvements in AIC value and accuracy. Although interaction effects are inherently imposed through the link function, in this work interaction terms have been included into generalized linear model for theoretical reasons since parameter-based interactions provide interactive effects that are different from the variety introduced by the link function identity.

(vi) For evaluating the quality of fit of the final model, several diagnostic statistical techniques had been applied [8]. Influence measures were analyzed considering Pearson residuals plotted against the fitted values (predicted values) and the Normal Q-Q plot of Standardized residuals, which can detect lack of fit, in fact if residuals present some curvilinear trend it could be the sign of a bad quality of the model. From the Normal Q-Q plot, it could be assessed if the normal distribution of residuals is influenced by several measures. For large dataset the normality assumption is not crucial but the presence of points diverging from linearity would indicate a long-tailed error [9]. In a manual and iterative way, single cases with the most relevant distance from linearity had been first checked by specialists and then removed from the dataset. The final model has been repeatedly updated without influence cases until a stop criterion (significance of each predictor has a p-value >0.01) had not been reached.

(vii) The final generalized linear model has been applied on POI and PCOS dataset. The hypothesis was that, in the case of POI, the ovarian age is greater than the chronological age and in the case of PCOS the ovarian age is lesser than the chronological age.

**References for detailed statistical analysis**

1. McCullagh P & Nelder JA. Generalized Linear Models. 2nd Edition.Chapman & Hall/CRC Monographs on Statistics & Applied Probability; 1989.
2. Breslow NE. Generalized linear models: checking assumptions and strengthening conclusions. Statistica Applicata. 1996;8:23-41.
3. Dormann CF, Elith J, Bacher S, Buchmann C, Carl G, Carré G & Lautenbach S. Collinearity: a review of methods to deal with it and a simulation study evaluating their performance. Ecography. 2013;36:27-46.
4. Akaike H. A new look at the statistical model identification. IEEE Transactions on Automatic Control. 1976;19:716-723.
5. Burnham KP, Anderson DR. Model Selection and Multimodel Inference: A Practical Information-Theoretic Approach. 2nd ed. Springer-Verlag; 2002
6. Stone M. An asymptotic equivalence of choice of model by cross-validation and Akaike's criterion.Journal of the Royal Statistical Society. Series B Methodological. 1977: 44-47.
7. Shao J. An asymptotic theory for linear model selection. Statistica Sinica. 1997;7:221-242.
8. Faraway JJ. Extending the Linear Model with R: Generalized Linear, Mixed Effects and Nonparametric Regression Models. CRC press; 2004.
9. Glosup J. Generalized Linear Models: An Applied Approach. Technometrics. 2005;47: 232-232.

**Supplementary tables**

**Supplementary tables**

**Table S1 Summary of the Generalized Linear Model (GzLM#2**), updated from GzLM#2* by removing 7 influence cases (645 HCs), resulting in the overall best model.**

| ***GzLM#2***** | ***Estimate*** | | | ***Std. Error*** | ***z value*** | | | ***Pr(>\|z\|)*** |  |  |
| --- | --- | --- | --- | --- | --- | --- | --- | --- | --- | --- |
| (Intercept) | 48.04723 | | | 1.23174 | 39.008 | | | < 2e-16 | *** |  |
| AMH | -3.13652 | | | 0.36981 | -8.481 | | | < 2e-16 | *** |  |
| FSH | 0.07391 | | | 0.03754 | 1.969 | | | 0.048979 | * |  |
| AFC | -0.776596 | | | 0.089375 | -8.689 | | | < 2e-16 | *** |  |
| FI | -0.11245 | | | 0.03085 | -3.645 | | | 0.000267 | *** |  |
| VI | 0.24710 | | | 0.09785 | 2.525 | | | 0.011560 | * |  |
| AMH:AFC | 0.102314 | | | 0.102314 | 5.439 | | | 5.34e-08 | *** |  |
| FSH:AFC | 0.019927 | | | 0.005097 | 3.909 | | | 9.25e-05 | *** |  |
|  | |  |  | | |  | **AIC: 3875.2** | | | |

*Signif. codes: 0 '***' 0.001 '**' 0.01 '*' 0.05 '.' 0.1 ' '*

**Table S2 Summary of the Generalized Linear Model (GzLM#1), built using all the eight variables and without interaction between them (652 HC).**

| ***GzLM#1*** | ***Estimate*** | | ***Std. Error*** | | ***z value*** | | | ***Pr(>\|z\|)*** |  |
| --- | --- | --- | --- | --- | --- | --- | --- | --- | --- |
| (Intercept) | 44.981 | | 1.27 | | 35.402 | | | < 2e-16 | *** |
| AMH | -1.466 | | 0.198 | | -7.395 | | | 1.41e-13 | *** |
| FSH | 0.209 | | 0.028 | | 7.527 | | | 5.19e-14 | *** |
| E2 | -0.007 | | 0.014 | | -0.526 | | | 0.599218 |  |
| AFC | -0.369703 | | 0.057791 | | -6.397 | | | 1.58e-10 | *** |
| FI | -0.125 | | 0.032 | | -3.885 | | | 0.000102 | *** |
| VI | 0.292 | | 0.1040 | | 2.820 | | | 0.004796 | ** |
| VFI | -0.037 | | 0.162 | | -0.229 | | | 0.818525 |  |
| Volume | -0.065 | | 0.126 | | -0.515 | | | 0.606862 |  |
|  | |  | |  | |  | **AIC: 3987.3** | | |

Signif. codes: 0 '***' 0.001 '**' 0.01 '*' 0.05 '.' 0.1 ' '

**Table S3 Summary of the Generalized Linear Model (GzLM#2), built using five variables and without interaction between them (652 HC).**

| ***GzLM#2*** | ***Estimate*** | ***Std. Error*** | | | ***z value*** | | ***Pr(>\|z\|)*** |  |  |
| --- | --- | --- | --- | --- | --- | --- | --- | --- | --- |
| (Intercept) | 44.47730 | 1.065 | | | 41.760 | | < 2e-16 | *** |  |
| AMH | -1.469 | -0.198 | | | -7.430 | | 1.09e-13 | *** |  |
| FSH | 0.215 | 0.026 | | | 8.231 | | 2e-16 | *** |  |
| AFC | -0.37135 | 0.05691 | | | -6.525 | | 6.81e-11 | *** |  |
| FI | -0.133 | 0.030 | | | -4.347 | | 1.38e-05 | *** |  |
| VI | 0.286 | 0.095 | | | 2.990 | | 0.00279 | ** |  |
|  |  | |  |  | | **AIC: 3981.9** | | | |

Signif. codes: 0 '***' 0.001 '**' 0.01 '*' 0.05 '.' 0.1 ' '

**Table S4. Summary of the Generalized Linear Model (GzLM#2*), built using five variables and considering main interactions between them (652 HC).**

| ***GzLM#2**** | ***Estimate*** | ***Std. Error*** | ***z value*** | ***Pr(>\|z\|)*** |  |
| --- | --- | --- | --- | --- | --- |
| (Intercept) | 47.82708 | 1.22988 | 38.888 | < 2e-16 | *** |
| AMH | -2.95335 | 0.36557 | -8.079 | 6.55e-16 | *** |
| FSH | 0.08639 | 0.03756 | 2.300 | 0.021453 | * |
| AFC | -0.735481 | 0.088708 | -8.291 | < 2e-16 | *** |
| FI | -0.12246 | 0.03068 | -3.992 | 6.56e-05 | *** |
| VI | 0.23827 | 0.09779 | 2.436 | 0.014833 | * |
| AMH:AFC | 0.095676 | 0.018775 | 5.096 | 3.47e-07 | *** |
| FSH:AFC | 0.018417 | 0.005084 | 3.622 | 0.000292 | *** |
|  |  |  |  | **AIC: 3955.2** | |

Signif. codes: 0 '***' 0.001 '**' 0.01 '*' 0.05 '.' 0.1 ' '

**Table S5 Summary of the seven cases removed from model GzLM#2* due to their influence on non-normality of residuals.**

| **PatID** | **OvAge** | **Age**  ***[years]*** | **AMH**  ***[ng/ml]*** | **FSH**  ***[mlU/ml]*** | **E_2_**  ***[pg/ml]*** | **AFC** | **FI** | **VI** | **VFI** | **Volume** |
| --- | --- | --- | --- | --- | --- | --- | --- | --- | --- | --- |
| **156** | 25.24 | 38 | 4.4 | 2.89 | 54.7 | 24.0 | 34.0 | 2.5 | 1.5 | 5.9 |
| **216** | 40.97 | 27 | 0.91 | 9.15 | 46.18 | 4.0 | 27.0 | 1.1 | 0.5 | 5.0 |
| **453** | 26.84 | 42 | 3.11 | 5.0 | 18.86 | 26.0 | 28.0 | 0.8 | 0.3 | 11.0 |
| **460** | 27.25 | 40 | 5.06 | 6.14 | 37.66 | 16.0 | 33.0 | 0.4 | 0.2 | 6.9 |
| **463** | 35.76 | 23 | 1.44 | 7.17 | 61.79 | 12.0 | 25.0 | 0.9 | 0.1 | 4.4 |
| **476** | 25.90 | 40 | 5.97 | 5.2 | 38.89 | 12.0 | 34.0 | 1.0 | 0.8 | 4.1 |
| **500** | 36.48 | 22 | 1.59 | 8.4 | 31.65 | 10.0 | 26.0 | 0.4 | 0.1 | 7.8 |
|  |  |  |  |  |  |  |  |  |  |  |
| **Min** |  | 22.0 | 0.91 | 2.89 | 18.86 | 4.0 | 25.0 | 0.4 | 0.1 | 4.1 |
| **Max** |  | 42.0 | 5.97 | 9.15 | 61.79 | 26.0 | 34.0 | 2.5 | 1.5 | 11.0 |
| **Mean** |  | 33.14 | 3.21 | 6.28 | 41.39 | 14.86 | 29.57 | 1.01 | 0.5 | 6.44 |
| **Std. dev.** |  | 8.76 | 1.98 | 2.15 | 14.37 | 7.82 | 3.95 | 0.71 | 0.51 | 2.41 |

**Table S6 Summary of 29 POI subjects, including fitted (predicted) values for age by model GzLM#2**.**

| **PatID**  **POI** | **OvAge** | **Age**  ***[years]*** | **AMH**  ***[ng/ml]*** | **FSH**  ***[mlU/ml]*** | **E_2_**  ***[pg/ml]*** | **AFC** | **FI** | **VI** | **VFI** | **Volume** |
| --- | --- | --- | --- | --- | --- | --- | --- | --- | --- | --- |
| 50 | 45.72 | 34 | 0.2 | 31.0 | 13.5 | 12.0 | 28.0 | 16.0 | 4.0 | 4.0 |
| 125 | 54.93 | 26 | 0.0 | 84.58 | 15.03 | 2.0 | 11.0 | 0.2 | 0.2 | 1.0 |
| 126 | 56.59 | 39 | 0.0 | 100.5 | 32.18 | 2.0 | 13.0 | 0.5 | 0.3 | 1.0 |
| 146 | 50.75 | 39 | 0.0 | 53.85 | 5.0 | 2.0 | 18.0 | 0.6 | 0.2 | 2.2 |
| 147 | 47.05 | 40 | 0.0 | 31.66 | 44.47 | 2.0 | 19.0 | 0.9 | 0.7 | 5.1 |
| 149 | 49.95 | 39 | 0.0 | 42.99 | 16.25 | 2.0 | 15.0 | 1.0 | 0.9 | 3.2 |
| 157 | 52.12 | 40 | 0.0 | 64.74 | 20.05 | 2.0 | 17.0 | 0.7 | 0.5 | 4.0 |
| 176 | 52.30 | 40 | 0.0 | 76.48 | 5.0 | 2.0 | 31.0 | 2.4 | 0.9 | 6.3 |
| 179 | 44.79 | 40 | 0.0 | 31.49 | 44.6 | 4.0 | 41.0 | 4.7 | 2.69 | 3.3 |
| 180 | 48.25 | 40 | 0.0 | 43.25 | 26.3 | 4.0 | 38.0 | 3.8 | 1.7 | 4.8 |
| 181 | 51.16 | 34 | 0.0 | 54.38 | 16.24 | 6.0 | 26.0 | 0.7 | 0.2 | 3.1 |
| 184 | 56.12 | 39 | 0.0 | 87.16 | 5.0 | 4.0 | 21.0 | 0.6 | 0.2 | 3.0 |
| 227 | 52.88 | 40 | 0.0 | 76.12 | 24.31 | 2.0 | 22.0 | 0.8 | 0.6 | 4.2 |
| 247 | 49.79 | 40 | 0.0 | 48.67 | 18.76 | 2.0 | 21.0 | 0.5 | 0.1 | 2.9 |
| 254 | 46.54 | 37 | 0.0 | 30.24 | 36.7 | 4.0 | 27.0 | 0.6 | 0.5 | 4.7 |
| 258 | 56.40 | 40 | 0.0 | 100.4 | 38.29 | 2.0 | 15.0 | 0.7 | 0.2 | 3.4 |
| 276 | 54.28 | 39 | 0.0 | 89.15 | 27.32 | 2.0 | 23.0 | 1.0 | 1.0 | 4.7 |
| 303 | 49.17 | 36 | 0.0 | 50.21 | 47.49 | 2.0 | 29.0 | 0.9 | 0.3 | 4.9 |
| 343 | 52.84 | 37 | 0.0 | 76.33 | 21.27 | 2.0 | 21.0 | 0.1 | 0.1 | 4.1 |
| 353 | 52.37 | 38 | 0.0 | 64.99 | 37.38 | 2.0 | 15.0 | 0.7 | 0.2 | 3.4 |
| 365 | 57.27 | 40 | 0.0 | 94.7 | 0.0 | 4.0 | 21.0 | 0.6 | 0.2 | 2.1 |
| 382 | 45.60 | 39 | 0.09 | 31.11 | 33.01 | 8.0 | 21.0 | 1.0 | 0.8 | 1.6 |
| 458 | 50.70 | 40 | 0.0 | 66.65 | 37.45 | 2.0 | 32.0 | 0.9 | 0.3 | 5.4 |
| 470 | 45.61 | 37 | 0.05 | 31.77 | 58.81 | 4.0 | 23.0 | 0.2 | 0.1 | 10.0 |
| 475 | 47.47 | 36 | 0.0 | 31.07 | 23.42 | 4.0 | 21.0 | 0.5 | 0.2 | 2.3 |
| 528 | 51.80 | 40 | 0.0 | 67.29 | 10.16 | 2.0 | 22.0 | 0.5 | 0.2 | 2.1 |
| 579 | 47.54 | 35 | 0.03 | 38.7 | 36.42 | 2.0 | 20.0 | 0.5 | 0.4 | 4.0 |
| 633 | 44.73 | 40 | 0.07 | 33.14 | 66.69 | 4.0 | 19.0 | 0.4 | 0.3 | 3.8 |
| 667 | 53.65 | 32 | 0.0 | 81.07 | 0.0 | 12.0 | 19.0 | 0.3 | 0.2 | 3.2 |

**Table S7 Summary of 29 PCO subjects, including fitted (predicted) values for age by model GzLM#2**. In red five cases in which the prediction of the ovarian age failed.**

| **PatID**  **PCO** | **OvAge** | **Age**  ***[years]*** | **AMH**  ***[ng/ml]*** | **FSH**  ***[mlU/ml]*** | **E_2_**  ***[pg/ml]*** | **AFC** | **FI** | **VI** | **VFI** | **Volume** |
| --- | --- | --- | --- | --- | --- | --- | --- | --- | --- | --- |
| 108 | 26.21 | 37 | 7.01 | 5.9 | 40.7 | 30.0 | 24.0 | 2.7 | 0.7 | 6.1 |
| 285 | 23.80 | 25 | 8.22 | 9.15 | 38.6 | 20.0 | 40.0 | 1.7 | 0.9 | 7.8 |
| 297 | 23.75 | 30 | 5.65 | 5.3 | 56.87 | 24.0 | 45.0 | 1.3 | 1.0 | 7.9 |
| 298 | 24.92 | 31 | 8.06 | 9.22 | 33.74 | 28.0 | 47.0 | 1.1 | 1.0 | 7.0 |
| 306 | 26.86 | 29 | 4.52 | 5.21 | 31.31 | 14.0 | 42.0 | 1.1 | 1.0 | 7.1 |
| 317 | 24.66 | 27 | 8.43 | 7.62 | 78.1 | 26.0 | 37.0 | 1.9 | 0.9 | 6.2 |
| 329 | 24.81 | 29 | 9.21 | 7.89 | 65.42 | 28.0 | 38.0 | 1.2 | 1.0 | 8.1 |
| 347 | 24.38 | 19 | 9.34 | 7.49 | 63.22 | 28.0 | 41.0 | 2.0 | 1.9 | 8.1 |
| 368 | 27.54 | 18 | 2.49 | 5.02 | 39.62 | 22.0 | 37.0 | 1.5 | 1.2 | 4.0 |
| 376 | 24.09 | 29 | 7.16 | 5.28 | 35.66 | 22.0 | 31.0 | 1.0 | 0.9 | 6.1 |
| 390 | 24.98 | 25 | 6.73 | 7.39 | 76.81 | 28.0 | 41.0 | 1.8 | 1.1 | 5.9 |
| 391 | 22.91 | 21 | 4.55 | 5.47 | 39.75 | 36.0 | 39.0 | 1.6 | 1.5 | 5.3 |
| 411 | 25.56 | 30 | 5.63 | 7.81 | 26.58 | 40.0 | 36.0 | 1.8 | 0.7 | 9.1 |
| 412 | 25.00 | 29 | 5.16 | 5.99 | 49.24 | 32.0 | 40.0 | 5.4 | 2.2 | 10.7 |
| 428 | 23.80 | 28 | 7.96 | 7.94 | 47.92 | 20.0 | 35.0 | 0.6 | 0.2 | 6.1 |
| 442 | 26.86 | 31 | 5.69 | 8.93 | 36.87 | 22.0 | 35.0 | 1.2 | 0.2 | 8.3 |
| 444 | 25.08 | 27 | 8.53 | 6.55 | 33.29 | 32.0 | 36.0 | 0.4 | 0.2 | 11.4 |
| 461 | 25.29 | 26 | 4.52 | 5.0 | 39.29 | 30.0 | 23.0 | 0.3 | 0.1 | 14.0 |
| 477 | 25.42 | 31 | 7.01 | 6.26 | 61.1 | 32.0 | 31.0 | 1.2 | 1.0 | 7.4 |
| 518 | 23.72 | 29 | 7.32 | 4.29 | 27.6 | 36.0 | 37.0 | 1.6 | 1.0 | 6.7 |
| 522 | 22.75 | 21 | 6.94 | 5.31 | 64.73 | 24.0 | 47.0 | 1.7 | 1.2 | 8.4 |
| 571 | 24.06 | 29 | 7.81 | 5.9 | 30.36 | 36.0 | 49.0 | 2.2 | 1.8 | 8.0 |
| 621 | 25.67 | 33 | 7.16 | 5.88 | 41.7 | 32.0 | 29.0 | 2.3 | 1.9 | 6.0 |
| 640 | 25.31 | 29 | 7.7 | 7.39 | 55.62 | 32.0 | 42.0 | 2.1 | 1.9 | 7.3 |
| 641 | 25.94 | 28 | 9.06 | 5.99 | 69.05 | 36.0 | 39.0 | 2.2 | 2.0 | 8.1 |
| 649 | 32.12 | 21 | 14.2 | 3.64 | 32.13 | 44.0 | 48.0 | 3.2 | 2.9 | 10.1 |
| 652 | 25.00 | 29 | 9.03 | 7.55 | 51.74 | 28.0 | 37.0 | 2.2 | 1.9 | 7.5 |
| 666 | 24.30 | 24 | 6.78 | 5.96 | 43.2 | 28.0 | 40.0 | 2.3 | 2.0 | 7.2 |
| 670 | 24.50 | 31 | 6.53 | 5.89 | 21.02 | 28.0 | 40.0 | 3.0 | 2.2 | 8.0 |
